# Liver Tumor Segmentation from MR Images Using 3D Fast Marching Algorithm and Single Hidden Layer Feedforward Neural Network

**DOI:** 10.1155/2016/3219068

**Published:** 2016-08-14

**Authors:** Trong-Ngoc Le, Pham The Bao, Hieu Trung Huynh

**Affiliations:** ^1^Faculty of Information Technology, Industrial University of Ho Chi Minh City, 12 Nguyen Van Bao, Go Vap District, Ho Chi Minh City, Vietnam; ^2^Faculty of Information Technology, University of Science, 227 Nguyen Van Cu, District 5, Ho Chi Minh City, Vietnam; ^3^Faculty of Mathematics and Computer Science, University of Science, 227 Nguyen Van Cu, District 5, Ho Chi Minh City, Vietnam

## Abstract

*Objective*. Our objective is to develop a computerized scheme for liver tumor segmentation in MR images.* Materials and Methods*. Our proposed scheme consists of four main stages. Firstly, the region of interest (ROI) image which contains the liver tumor region in the T1-weighted MR image series was extracted by using seed points. The noise in this ROI image was reduced and the boundaries were enhanced. A 3D fast marching algorithm was applied to generate the initial labeled regions which are considered as teacher regions. A single hidden layer feedforward neural network (SLFN), which was trained by a noniterative algorithm, was employed to classify the unlabeled voxels. Finally, the postprocessing stage was applied to extract and refine the liver tumor boundaries. The liver tumors determined by our scheme were compared with those manually traced by a radiologist, used as the “ground truth.”* Results*. The study was evaluated on two datasets of 25 tumors from 16 patients. The proposed scheme obtained the mean volumetric overlap error of 27.43% and the mean percentage volume error of 15.73%. The mean of the average surface distance, the root mean square surface distance, and the maximal surface distance were 0.58 mm, 1.20 mm, and 6.29 mm, respectively.

## 1. Introduction

The hepatic cell carcinoma (HCC) is one of the most common cancers and rapidly growing worldwide. It was estimated that up to 90% of patients with liver cancer would be dead within five years of diagnosis [1]. The early detection and the treatment response evaluation of patients with the liver cancer are very important to improve the survival rate. The basic criterion to evaluate the tumor response to the treatment is the tumor size. There are some criteria for tumor size including one-, bi-, or tridimensional measurement [2–5]. However, clinical researches have shown that the volume measurement (3D) can give the best reflection of the tumor response [3–5]. The liver tumor volumetry requires the tumor segmentation. Traditionally, this task can be performed by manually tracing the tumor regions on slices. It is tedious and time-consuming. In addition, the volume of manual delineations is subjective; it was estimated that the intra- and interobserver variability are about 8% for liver tumors [6]. Hence, it is crucial to investigate in the computerized scheme for liver tumor segmentation.

Some computerized schemes have been developed for the liver tumor segmentation on CT images. These methods include the watershed like the paintbrush algorithm [7], deformable models [8], and level-set and active contour techniques. Multiple thresholding and adaptive techniques for liver tumor segmentation from CT images also were proposed by researchers [9–11]. Wong et al. [12] proposed a region growing technique based on knowledge-constraints for liver tumor segmentation in each slice. This technique demonstrated an average volume difference of 24.2% in tumor segmentation. Ben-Dan and Shenhav [13] developed an approach for liver tumor segmentation based on the active contour using a weighted function of the probability of each pixel. An approach for liver tumor segmentation based on Bayesian classification and the active contour method was proposed by Taieb et al. [14]. This approach can obtain an average volume difference of 44.2%. Smeets et al. [15] proposed an approach based on the combination of the level-set method and the spiral-scanning technique. In this method, users place a point approximately in the middle of the liver tumor and specify a maximal radius by placing other points surrounding the tumor. Next, the spiral-scanning technique was applied to generate the initial surface for the level-set segmentation. A method for the liver tumor segmentation by applying the minimal surfaces and Markov random fields was developed by Stawiaski et al. [16]. Some techniques using machine learning methods also were developed [17–21]. Li et al. [17] developed a technique for liver tumor segmentation based on machine learning using the intensity profiles of the liver tumors. This method is less accurate for segmenting the tumors with irregular boundaries due to biasing to the blob-like ones. Zhou et al. [18] developed a scheme, in which the tumor region from a single slice was extracted by using support vector machine (SVM). This region was propagated and applied to classify voxels in other slices which contain the tumor. A scheme for 3D liver tumor segmentation based on the random walker has been proposed by Jolly and Grady [19]. The user-defined seed point is required, and the additional seed points are generated from 2D fuzzy-connectedness segmentation of a slice containing the seed point. Freiman et al. [20] developed an approach for liver tumor segmentation from CTA using SVM, in which the features include the mean, the standard deviation, and the minimum and the maximum intensity values in a predefined window surrounding the voxel. Huang et al. [21] demonstrated an approach for liver tumor segmentation based on extreme learning machine. The features are generated from the mean, variance, intensity, intensity power, entropy, intensity cooccurrence, Law's texture, and sum and difference histogram.

In comparison with CT images, the number of approaches for liver tumor segmentation on MR images is limited, while the increasing use of liver MRI as a single exam for liver disease leads to imperative demands for investigating researches in automatic MRI liver tumor volumetry.

Developing the computerized scheme for liver tumor segmentation in MR images is a challenging task. The images have a low gradient response. The liver tumors generally have different shapes. The gray values of tumor depend on several factors including the tumor type, the image acquisition, and the contrast injection. In this study, we focus on a very challenging task of the liver tumor segmentation in abdominal MR images. The proposed method utilizes the local information in the liver; it combines the 3D fast marching algorithm and the neural network. The region of interest (ROI) surrounding the tumor is established to improve the performance of generating the initial regions as well as reduce the computational requirements.

## 2. Materials and Methods

**Figure 1:**
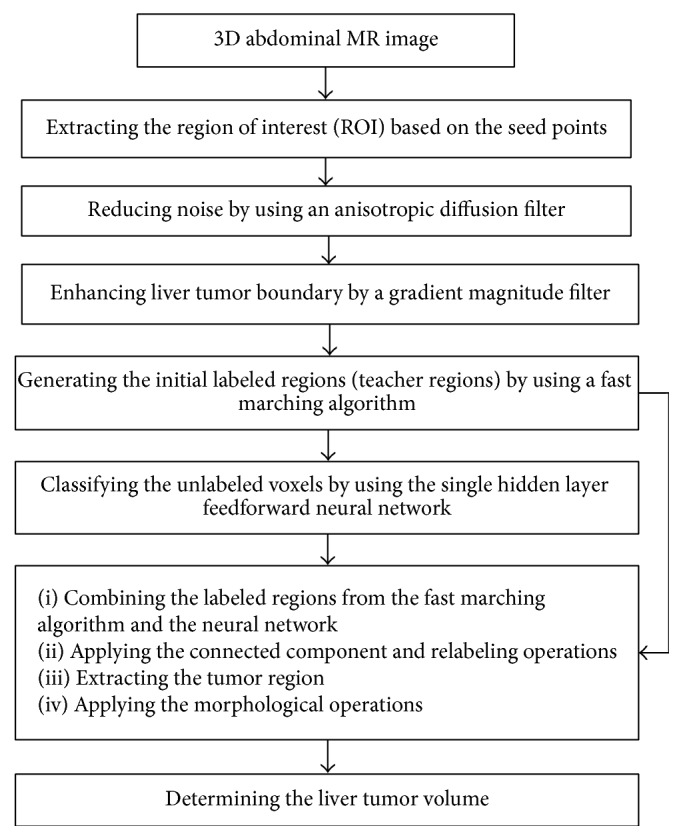
Overview of the proposed scheme for liver tumor segmentation.

Our proposed scheme for liver tumor segmentation is described in Figure 1. It consists of four main stages: preprocessing, generating the initial labeled regions, classifying the unlabeled voxels using the single hidden layer feedforward neural network (SLFN), and postprocessing.

### 2.1. Preprocessing Stage

Firstly, users can choose the seed points inside and outside the tumor. The 3D region of interest (ROI), *I*_*R*_^0^(*x*, *y*, *z*), involving the tumor is determined based on the seed points. This ROI is passed to reduce noise and enhance the liver tumor structures by using an anisotropic diffusion algorithm. This algorithm is handled by a modified curvature diffusion equation given by

(1) $$\begin{array}{c} \frac{\partial I_{R}}{\partial t}=\left|\;\nabla I_{R}\;\right|\nabla \cdot c\left(\;\left|\;\nabla I_{R}\;\right|\;\right)\frac{\nabla I_{R}}{\left|\nabla I_{R}\right|}, \end{array}$$

where *I*_*R*_(·, *t*) is the image function with the initial image at *t* = 0 given by *I*_*R*_(·, *t* = 0) = *I*_*R*_^0^(·) and *c*(·) is the diffusion coefficient which controls the sensitivity of the edge contrast. This algorithm smooths noise in the image while preserving the major structures including the boundaries. The noise-reduced image is then passed through a Gaussian gradient magnitude filter to generate *I*_*G*_ which enhances the boundaries. The gradient magnitude image is determined by

(2) $$\begin{array}{c} I_{M}=\sqrt{{\left(\frac{\partial I_{G}}{\partial x}\right)}^{2}+{\left(\frac{\partial I_{G}}{\partial y}\right)}^{2}+{\left(\frac{\partial I_{G}}{\partial z}\right)}^{2}}. \end{array}$$

The gradient magnitude image is then employed to produce the edge potential image by using a sigmoid function given by

(3) $$\begin{array}{c} I_{P}=\frac{1}{1+e^{\left\{-\left(I_{M}-\beta \right)/\alpha \right\}}}, \end{array}$$

where *α* and *β* are parameters specifying the range and center, respectively, of the intensity to be enhanced. In this scheme, *α* and *β* were −3.5 and 8.0, respectively. The edge potential image is used as a speed function for a fast marching algorithm to generate the initial labeled regions.

### 2.2. Generating the Initial Labeled Regions

The main goal in this stage is to generate the labeled regions or teacher regions by using a fast marching algorithm. This algorithm is based on the numerical solution of the Eikonal equation given by

(4) $$\begin{array}{c} \left|\;\nabla T\;\right|F=1, \end{array}$$

where *T* is an arrival time function and *F* is a speed function which is obtained from the potential image, *I*_*P*_. The algorithm requires the initial seed points which correspond to the initial location (*T* = 0). These seed points (1-2 points inside the tumor and 1–3 points outside the tumor) in our scheme are placed by a radiologist. In the first step of the algorithm, the grid points from the entire image are categorized into three classes. The initial seed points are classed as* Known*. The neighbors of the* Known* points are classed as* Trial*, and their arrival time is computed by using the first-order scheme (see (4)). Other points are classified as* Far* with the infinity arrival time. The algorithm propagates the information in one way from the smaller values to the larger values of *T*. The point **q** with the smallest arrival time in the* Trial* list is chosen and moved to the* Known* one. The neighbors of **q** are moved to the* Trial* list and the arrival time is recomputed by using the first-order scheme. This iterative process is terminated when the maximum number of iterations is met. The salient point of this algorithm is that the heap data structure is used to speed up locating the* Trial* point with the smallest *T* value. The output of the fast marching algorithm is a time-crossing map image which indicates the time travelling to each point. The labeled regions are generated by
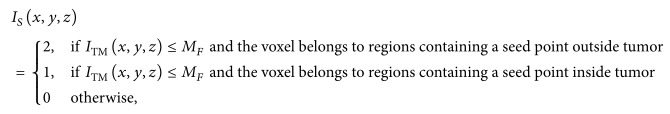
(5)where *M*_*F*_ is the maximum number of iterations for the fast marching algorithm and *I*_TM_ is the time-crossing map image. The points with the values of 1 or 2 in the image *I*_*S*_ are considered to be labeled, otherwise considered to be unlabeled because they were not treated from the fast marching algorithm. Due to variations of intensity in tumors, the labeled regions could not form the rough shape of the liver tumor. However, they can be considered as teacher regions for training the neural network to classify the unlabeled points.

### 2.3. Classifying the Unlabeled Voxels

The labeled points are categorized into two classes, tumor or nontumor corresponding to the labels of 1 or 2, respectively. Now, we have to classify the unlabeled points which are not treated by the fast marching algorithm. The neural network is one of the powerful tools in biology and medical data analysis applications [22, 23]. Hence, in this study we utilized the neural network to categorize the unlabeled points.

Several network architectures have been developed; however a single hidden layer feedforward neural network (SLFN) can form the decision boundaries with arbitrary shapes if the activation function is chosen properly. A typical architecture of SLFN consists of an input layer, a hidden layer with *K* units, and an output layer with *M* units. In this study, the SLFN handles the input gray levels directly. The inputs to the SLFN comprise the normalized voxel in ROI image (*I*_*R*_) and its spatially adjacent normalized voxels within the local window *L*_*W*_. An input pattern corresponding to *I*_*R*_(*x*, *y*, *z*) is determined by

(6) pxyz=−1+2IRx−i,y−j,z−k−IminImax−Imin ∣ i,j,k∈LW,

where *I*_min_, *I*_max_ are the minimum and maximum voxel values, respectively, in *I*_*R*_. The output of the SLFN which corresponds to the center voxel in the local window is the value indicating either tumor or nontumor, represented by

(7) $$\begin{array}{c} o_{xyz}=\overset{K}{\underset{k=1}{\sum }}a_{k}\varphi \left(\;p_{xyz}\cdot w_{k}+b_{k}\;\right), \end{array}$$

where **w**_*k*_ is the input weights connecting from the input layer to the *k*th hidden unit, *b*_*k*_ is its bias, **a**_*k*_ = [*a*_*k*1_, *a*_*k*2_,…,*a*_*kM*_]^*T*^ is the output weights connecting from the *k*th hidden unit to the output layer, and *φ*(·) is the activation function of the hidden layer which was a sigmoidal function in this study. The activation function of the output layer was an identify function.

One of the important issues in the SLFN is to train the network to determine the network weights **w**, *b*, and **a**. Traditionally, training the neural network could be performed by employing the backpropagation algorithm. This algorithm has some limitations such as slow convergence, local minima, overfitting, or improper learning rate. Although there are several improvements to overcome the problems of backpropagation algorithm, up to now, the training algorithms based on the gradient descent approach are still slow due to many iterative steps. In this study, we employed an effective training algorithm for SLFN called extreme learning machine (ELM) and its improvements [24–27].

The desired output corresponding to the input pattern **p**_*xyz*_ is obtained from the teacher regions in *I*_*S*_, represented by

(8) $$\begin{array}{c} t_{xyz}=\left\{\begin{array}{cc} {\left(\begin{array}{cc} 0 & 1 \end{array}\right)}^{T} & \text{if }I_{S}\left(x,y,z\right)=1\\ {\left(\begin{array}{cc} 1 & 0 \end{array}\right)}^{T} & \text{if }I_{S}\left(x,y,z\right)=2. \end{array}\right. \end{array}$$

Assume that there are *n* labeled points in the teacher regions. The training set is given by *S* = {(**p**_*i*_, **t**_*i*_), *i* = 1,2,…, *n*}, where the input patterns **p**_*i*_ are obtained from *I*_*R*_ by using (6) and its corresponding desired output, **t**_*i*_, is obtained from the teacher regions in *I*_*S*_ by using (8). The error function to be minimized by training process is defined by

(9) $$\begin{array}{c} E=\overset{n}{\underset{i=1}{\sum }}\left\|\;o_{i}-t_{i}\;\right\|, \end{array}$$

where **o**_*i*_ is the actual output corresponding to the input pattern **p**_*i*_ (by using (7)). In the extreme learning machine, the training process can be modeled by finding the solution of a linear model given by **H** **A** = **T**, where **H** is the hidden layer output matrix of SLFN defined by

(10) H=φw1·p1+b1⋯φwK·p1+bK⋮⋱⋮φw1·pn+b1⋯φwK·pn+bK,T=t1t2⋯tnT,A=a1a2⋯aKT.

In the extreme learning machine, the biases and input weights are assigned by the random values, and the output weights are found by using the Moore-Penrose generalized inverse:

(11) $$\begin{array}{c} \overset{⌢}{A}=H^{†}T, \end{array}$$

where** H** ^†^ is the pseudoinverse of** H**. Several improvements of ELM have been developed by researchers [26, 27]. In order to make the system more stable, the regularization approaches have been proposed, in which a coefficient *λ* is added and the solution of** A** is given by

(12) $$\begin{array}{c} \overset{⌢}{A}={\left(\;H^{T}H+\lambda I\;\right)}^{-1}H^{T}T. \end{array}$$

These training algorithms are fast and can offer a good performance in many applications including medical data analysis.

### 2.4. Postprocessing

The classifying results from the SLFN for unlabeled regions are combined with the ones from the fast marching algorithm. A voxel belongs to tumor region if it is classified as tumor by either the fast marching algorithm or the SLFN. The combined results are then passed to the connected component and the relabeling filters. The region containing the tumor seed points is extracted and passed to remove small isolated artifacts and small holes by using the morphological operations. The liver tumor volume is calculated from the segmented tumor regions.

## 3. Experimental Results

### 3.1. Datasets

This study was approved by the institutional review board (IRB) of the Medic Medical Center. The datasets consist of 25 liver tumors which were obtained from 16 patients. Fifteen tumors were obtained from 10 patients by using the 1.5 T magnetic resonance imaging (MRI) scanners (Avanto, Siemens) at the Medic Medical Center, which is one of the largest diagnostic imaging centers in Vietnam. Informed consent was obtained from all patients. Postcontrast MR images were obtained by using the T1-weight volumetric interpolated breath-hold examination (VIBE) sequence. A flip angle of 10 degrees was used with the TE and TR of 2.38 and 4.74, respectively. The scanning parameters included collimation and reconstruction intervals ranging from 3.5 to 4 mm. Each MRI slice had a matrix size of 230 × 320 pixels with an in-plane pixel size ranging from 1.18 to 1.4 mm. The number of slices in each case ranged from 44 to 56. Ten other tumors were obtained from 6 patient cases which were extracted from The Cancer Imaging Archive (TCIA) [28].

A board-certified abdominal radiologist carefully manually traced the tumor contours on each slice which contains the liver tumor. The liver tumor volume was calculated by multiplying the areas of the manually traced regions in each slice by the reconstruction interval. The total tumor liver volume in each case was determined by the summation of the volumes in all of the slices. The times required to complete the manual contour tracing were recorded.

### 3.2. Evaluation Criteria

The liver tumor volumes obtained by our computerized approach were compared to the “ground-truth” manual volumes which were determined by the radiologist. The true positive (TP), false positive (FP), and false negative were calculated for detail analysis. The percentage volume error (*E*) for each computerized volume (*V*_*c*_) and the gold standard manual volume (*V*_*m*_) is determined by

(13) $$\begin{array}{c} E=\left|\;\frac{\left(V_{c}-V_{m}\right)}{V_{m}}\;\right|. \end{array}$$

The volumetric overlap error representing the fraction of the overlapping volume and the volume of two segmentation methods is given by

(14) $$\begin{array}{c} \text{VO}=1-\frac{\left|\text{TP}\right|}{\left|\text{TP}\right|+\left|\text{FP}\right|+\left|\text{FN}\right|}. \end{array}$$

Three surface distance metrics including the average symmetric surface distance (ASSD), the root mean square symmetric surface distance (RMSSD), and the maximal symmetric surface distance (MSSD) also were used to evaluate the global and the local disagreement between two segmentation methods.

### 3.3. Results and Discussions

**Figure 2:**
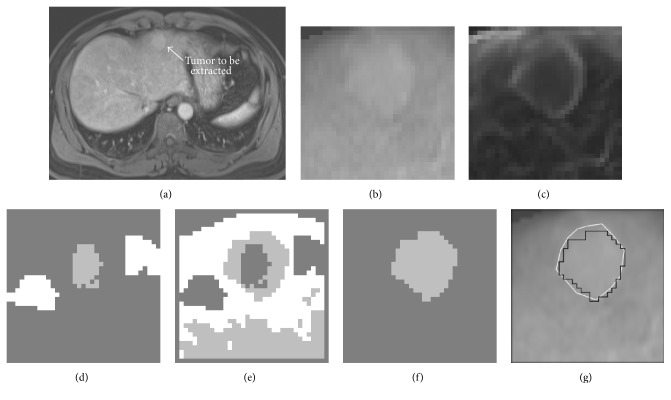
The intermediate results of the proposed scheme. (a) A slice of the original 3D image. (b) A slice of the 3D region of interest containing the liver tumor which was extracted from the original 3D MR image. (c) The edge image generated by applying the gradient magnitude filter. (d) The labeled regions generated by the fast marching algorithm and thresholding filter. (e) Unlabeled voxels were classified by using the SLFN. (f) The segmented liver tumor. (g) A comparison between the computerized liver tumor segmentation (black contour) and the “ground-truth” manual liver tumor segmentation (white contour).

The intermediate results of the proposed scheme for an example case were shown in Figure 2. An original 3D MR image was shown in Figure 2(a). The 3D region of interest (ROI) which was extracted from the original 3D MR image was shown in Figure 2(b). This ROI image was passed to reduce the noise by using an anisotropic diffusion algorithm. A gradient magnitude filter was applied to the reduced-noise image to enhance the boundaries and create an edge image, as shown in Figure 2(c). The edge potential image was generated, and then the fast marching algorithm and thresholding filter were applied to generate the image with the labeled regions, as shown in Figure 2(d). The labeled regions were employed to train the SLFN; this trained network was then used to classify voxels in the unlabeled regions as shown in Figure 2(e). The classification result from SLFN was combined with the one from the fast marching algorithm. The connected component and relabeling operations were applied, and the region which contains the seed points inside tumor was filtered out as shown in Figure 2(f). A comparison between the computerized liver tumor segmentation (black contour) and the “ground-truth” manual liver tumor segmentation (white contour) was illustrated in Figure 2(g). The liver tumor volume was computed from the segmented regions.

The average liver tumor volumes measured from two methods for two datasets, Medic Medical Center and TCIA, were shown in Table 1. For both datasets, the average tumor volume of the reference standard manual method was 15.36 ± 36.77 cm^3^ (range: 0.19–162.89 cm^3^), whereas the average tumor volume of the proposed computerized scheme was 13.88 ± 33.21 cm^3^ (range: 0.10–147.95 cm^3^). A comparison of the liver tumor segmentation on MR image between two methods was shown in Table 2. For both datasets, the overall mean of volumetric overlap error and the mean percentage volume error were 27.43% and 15.74%, respectively. The mean of average surface distance, root mean square surface distance, and maximal surface distance were 0.58 mm, 1.20 mm, and 6.29 mm, respectively.

It is not easy to directly compare the proposed method with existing methods in literature because of using different databases and quality measurements. Freiman et al. [20] evaluated the performance of their proposed scheme on CTA images, and it could obtain a volume overlap error and a volume difference of 33.8% and 22.6%, respectively. The mean of average surface distance, the RMS surface distance, and the maximal surface distance were 1.76 mm, 2.62 mm, and 13.73 mm, respectively. Smeets et al. [15] obtained the overlap error and the volume difference of 32.64% and 17.91%, respectively. The average, the RMS, and the maximum surface distance were 1.97 mm, 2.64 mm, and 10.13 mm, respectively. The method proposed by Huang et al. [21] could achieve the volume overlap error, the volume difference, the average surface distance, the RMS surface distance, and the maximal surface distance of 67.15%, 14.16%, 2.27 mm, 2.47 mm, and 8.46 mm, respectively; the performance of this scheme was evaluated on CT images.

There are several parameters to be adjusted in our proposed scheme. They were determined by empirical analysis. The number of hidden nodes in the SLFN was given by *K* = 200 and *λ* = 0.01; the local window size for extracting features (*L*_*W*_) was set to 3 × 3 × 3. The number of iterations for the fast marching algorithm was set to 15. All the parameters were fixed for all patients.

Some advanced schemes have applied the statistical models, used the intensity profiles of liver tumors, or used filters for the tumor segmentation. However, the actual tumors often differ from the simple models. There are tumors of various shapes and inhomogeneous tumors. In this study, the artificial neural network (ANN) was developed for accommodating the task of distinguishing the tumor voxels from the nontumor voxels. The network was trained by the subregions of the 3D MR image and operates on the voxel data directly, which is similar to the MTANN (massive training artificial neural network) [29]. However, unlike the original MTANN, the ANN proposed in this study was trained by the data from the 3D local information of each tumor, and the network was trained by a noniterative training algorithm which can overcome problems of the traditional training algorithms such as local minima, learning rate, and epochs. This training algorithm can offer a good performance with high learning speed in many different applications.

A limitation in this study is the number of patients. Our proposed scheme was evaluated in 16 patients with 25 tumors while other studies evaluated 5 patients with 10 tumors [20], 7 patients with 10 tumors [15], and 10 patients with 10 lesions [30]. In general, a small number of tumors and patients may limit the variations among them. In the future, we will need to increase the number of patients and tumors used for evaluation.

## 4. Conclusion

The increasing use of liver MRI as a single exam leads to imperative demands for investigating researches in the computerized MRI liver tumor segmentation. However, few studies have been reported for this challenging task. In this study, we developed a computerized scheme for liver tumor segmentation on MR images by employing the fast marching algorithm and the neural network, in which the neural network was trained by an effectively noniterative algorithm. The performance was evaluated in 25 tumors of 16 patients. Our experimental results have shown that the proposed method is accurate and efficient when compared to the manual ground-truth segmentation. Its accuracy also is comparable or better than the existing semiautomatic methods. With our proposed scheme, the time required for liver tumor segmentation is reduced significantly. Hence, it can be useful for radiologists for the liver tumor analysis on MR images.

## Figures and Tables

**Table 1:** Comparison of liver tumor volume between the computerized method and the manual method.

Dataset	Volume	Manual method (cc)	Computerized method (cc)
Medic Medical Center	Average	3.48	3.16
	SD	4.00	3.97
	Min	0.23	0.14
	Max	16.10	16.18

TCIA	Average	33.18	29.95
	SD	54.69	49.38
	Min	0.19	0.10
	Max	162.89	147.95

*Both*	Average	15.36	13.88
	SD	36.77	33.21
	Min	0.19	0.10
	Max	162.89	147.95

**Table 2:** Summary of the comparison results.

Dataset	Evaluation measure	Mean	SD	Min	Max
Medic Medical Center	Volumetric overlap error (%)	26.66	7.06	15.70	39.47
	Percentage volume error (%)	16.68	12.51	0.17	39.47
	Average surface distance (mm)	0.44	0.47	0.21	2.12
	RMS surface distance (mm)	0.97	0.97	0.53	4.40
	Maximal surface distance (mm)	4.84	4.89	1.68	21.18

TCIA	Volumetric overlap error (%)	28.57	10.89	13.71	47.89
	Percentage volume error (%)	14.32	15.81	0.21	47.89
	Average surface distance (mm)	0.79	0.73	0.14	2.66
	RMS surface distance (mm)	1.55	1.06	0.35	3.90
	Maximal surface distance (mm)	8.46	6.45	1.61	19.60

*Both*	Volumetric overlap error (%)	27.43	8.63	13.71	47.89
	Percentage volume error (%)	15.74	13.65	0.17	47.89
	Average surface distance (mm)	0.58	0.60	0.14	2.66
	RMS surface distance (mm)	1.20	1.03	0.35	4.40
	Maximal surface distance (mm)	6.29	5.73	1.61	21.18
